# Recent Advances in Tunable Metasurfaces and Their Application in Optics

**DOI:** 10.3390/nano13101633

**Published:** 2023-05-13

**Authors:** Alberto Santonocito, Barbara Patrizi, Guido Toci

**Affiliations:** 1 National Institute of Optics-National Research Council (INO-CNR), Via Madonna del Piano 10, 50019 Sesto Fiorentino, Italy; alberto.santonocito@ino.cnr.it (A.S.); guido.toci@ino.cnr.it (G.T.); 2 Department of Chemistry and Industrial Chemistry, Via G. Moruzzi 13, 56124 Pisa, Italy

**Keywords:** tunable metasurfaces, metalenses, meta-optics, electric tuning, optical tuning, magnetic tuning, temperature tuning, mechanical tuning, multiresponsive metasurfaces

## Abstract

Metasurfaces can be opportunely and specifically designed to manipulate electromagnetic wavefronts. In recent years, a large variety of metasurface-based optical devices such as planar lenses, beam deflectors, polarization converters, and so on have been designed and fabricated. Of particular interest are tunable metasurfaces, which allow the modulation of the optical response of a metasurface; for instance, the variation in the focal length of a converging metalens. Response tunability can be achieved through external sources that modify the permittivity of the materials constituting the nanoatoms, the substrate, or both. The modulation sources can be classified into electromagnetic fields, thermal sources, mechanical stressors, and electrical bias. Beside this, we will consider optical modulation and multiple approach tuning strategies. A great variety of tunable materials have been used in metasurface engineering, such as transparent conductive oxides, ferroelectrics, phase change materials, liquid crystals, and semiconductors. The possibility of tuning the optical properties of these metamaterials is very important for several applications spanning from basic optics to applied optics for communications, depth sensing, holographic displays, and biochemical sensors. In this review, we summarize the recent progress on electro-optical magnetic, mechanical, and thermal tuning of metasurfaces actually fabricated and experimentally tested in recent years. At the end of the review, a short section on possible future perspectives and applications is included.

## 1. Introduction

Metasurfaces consist of two-dimensional periodic arrangements of sub-wavelength scatterers, or meta-atoms placed on a lattice, that are able to modify the wavefront and/or the polarization distributions of an optical wave [1,2,3].

The optical function of a metasurface basically arises from scattering or absorption of the incident light field on a nanometer scale and the interaction can depend on the light polarization [4]. The scattering gives rise to reflection or to the modification of the properties of transmitted light.

Metasurfaces have shown promising applications in the field of optics, providing new opportunities for manipulating light in various applications, such as optical imaging, sensing, displays, and communication [5]. In addition, they can offer more efficient, cost-effective, and compact solutions compared with traditional optical components.

The physics governing metasurface functioning is based on the wave equation, which is used to calculate the propagation of waves in a medium. The Fresnel–Kirchhoff diffraction equation is used to determine the pattern of light diffraction [6], while Snell’s law of refraction can be used to calculate the refraction angle of light when it passes through a metalens, as well as how the light is focused [7]. The Gouy–Chapman equation is used to calculate the electrical field at the metalens surface, which is then used to determine how light is focused through the metalens [8]. Finally, the Fresnel equations are used to calculate the reflection and transmission of light through a metasurface [9].

The above-mentioned equations are commonly applied to predict the light propagation behaviour across metasurfaces. In this frame, metasurfaces have been experimentally realized for many application such as lenses [10,11], diffractive optical elements [12,13], and waveplates [14], as well as in unconventional functional optics such as polarimetric sensors [15], optical vortex converters [16], and beam steering [17]. In particular, metalenses have attracted attention because of their ultrathin and engineerable structures, which make them compact and thus highly integrable with respect to conventional single convex/concave lenses [18]. The first prototypes of metalenses, i.e., plasmonic metalenses, based on metallic meta-atoms [19], were limited by absorption losses and efficiency limits. In recent years, researchers focused on dielectric metalenses consisting of materials with a high refractive index and low losses [11,20], in which corrections for monochromatic and chromatic aberrations can be enabled by a single layer or a few layers of nanostructures. In general, the scatterers are designed as high contrast pillars made of meta-atoms with a large refractive index and low extinction coefficient depending on the specific spectral range. Titanium oxide (TiO_2_) [21], gallium nitride (GaN) [22], silicon nitride (SiN) [23], hydrogenated amorphous silicon (a-Si:H) [24], and SiO_2_ [25], among others, are widely used material candidates for the visible spectral range, while a-Si, gallium arsenide (GaAs) [26], gallium nitride (GaN) [27] aluminium nitride (AlN) [5], and litium niobate (LiNbO_3_) [28] are the most commonly used for near-infrared applications.

The computer modelling of metalens structures takes advantage of software packages using the finite-difference time-domain (FDTD) method [29], finite elements method (FEM) [30], or rigorous coupled wave analysis (RCWA) [31]. All of these methods solve Maxwell’s equations in either the time or frequency domain and return the same results in principle to obtain predictions that agree quantitatively with experiments. This is feasible for small systems such as periodic systems and metasurfaces that can be reduced into unit cells.

To fully exploit all of the possible geometries as well as the disordered structure, computer-aided inverse design of photonic devices or nano-structures has been taken into account by the scientific community [32,33,34]. Emerging deep learning techniques, especially artificial neural networks (ANN), provide a new perspective to realize complex functions based on a collection of connected units (neurons) that are arranged into different layers and connected through interlayer operations. By parameterizing the geometry as well as the optical spectrum, the connection between the metasurfaces and corresponding optical responses can be well learned and mathematically approximated by ANN [34,35,36].

While many efforts have focused on static metalenses, a further spreading of this technology into real-world applications requires the development of reconfigurable metalenses whose optical response can be adjusted in real time [37].

These tunable metasurfaces can be manipulated electrically, optically, mechanically, and thermally in order to allow a dynamic control on the outcoming wave [38].

Tunable metasurfaces can be used for a range of applications, such as wavefront shaping, polarization control [39], beam steering [40], retroreflectors [41], metalens [42], cloaking [43], optical encryption [44,45], and quantum information [46], among others.

They are also of particular interest in wireless communications. Digitally programmable metasurfaces have been used to encode signals in both spatial and spectral domains, allowing the transmission of digital messages to different users at different locations simultaneously [47]. Programmable metasurfaces, operating at terahertz frequencies, have also been created using arrays of complementary metal–oxide semiconductor (CMOS)-based chip tiles [48].

A particular class of tunable metasurfaces is represented by liquid crystal (LC)-based active metasurfaces. LC technology has become highly advanced after decades of development in LC-based devices such as LC displays (LCDs) and LC on silicon (LCoS) spatial light modulators (SLMs) [49,50]. LCs possess high optical birefringence that can be controlled by both the temperature and electric field [51,52].

Considering the enormous design versatility of metasurfaces in terms of geometries, materials, and dimensionality, it is clear that the fields of application will be more and more numerous in the coming years, especially if effective tuning mechanisms and simple and cheap production methods will be implemented. In this frame, in the last few years, several reviews focused on tunable metasurfaces have been presented [53,54,55,56].

In this review, we revise the recent literature to summarize all of the recent progress in tunable metasurfaces designed for optics applications. In this regard, in Section 2, we firstly point out the physical mechanisms that allow modifying the relative permittivity tensor, i.e., the optical properties of metamaterials when different kind of external stimuli are applied. This outline of the physical principles underlying the tuning mechanisms is useful to guide a comprehensive and systematic classification of the different approaches presented in the literature. Then, we summarize the recent progress on electric, optical, magnetic, mechanical, thermal, and multiple *stimuli* tuning of metasurfaces realized and experimentally tested in recent years. At the end of the review, a short section on the possible future perspectives and possible applications of tunable metasurfaces is included.

## 2. Metasurface Tuning Strategies

There are several ways to make a metasurface tunable. Some of the more popular methods consist of the application of an external magnetic field, an electrical bias, an optical stimulus, a temperature gradient, and mechanical stress factors. All of these methods allow for different modification of the relative permittivity tensor of the nanostructures, of the substrate, or of both. These modifications cause a change in the response of the metasurface when it interacts with an incident electromagnetic field.

**Figure 1:**
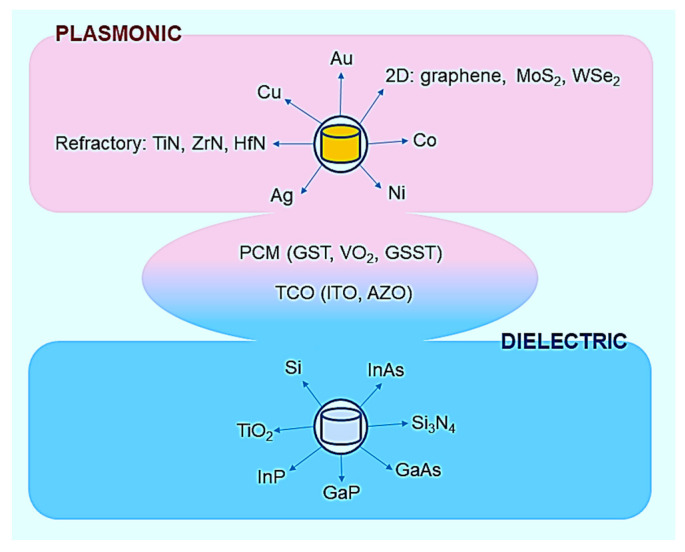
Metasurfaces can be divided into two general classes: plasmonic and dielectric metasurfaces. Particular materials such as phase change materials (PCMs) and transparent conducting oxides (TCOs) can reversibly change their properties between plasmonic and dielectric through an external stimulus, which may enable phase changes, or through carrier charge promotion in the conduction band.

The tuning strategies are strictly related to the meta-materials’ properties. Generally speaking, the metasurfaces can be classified as plasmonic or dielectric. Moreover, there are other materials such as phase change materials (PCMa) and transparent conducting oxides (TCOs) that can reversibly change their properties between plasmonic and dielectric through an external stimulus, which may enable phase changes (such as crystalline to amorphous or vice versa), or through carrier charge promotion in the conduction band (see Figure 1).

The different modulations that can be achieved using different stimuli of perturbations of the relative permittivity tensor describe the main perturbative effects due to the magnetic field, electrical stimuli (current or voltage), optical stimuli, temperature, and mechanical stressors, respectively.

Let us start with the magnetic field perturbation. The interaction between a magnetically polarized material such as Ni, Co, Cr, and YIG (yttrium iron garnet) and electromagnetic radiation results in a change in the permittivity tensor ε of the material. If the absorption losses can be neglected, ε is a Hermitian matrix. Thus, the permittivity tensor ε becomes anisotropic with complex out of diagonal elements ${\varepsilon^{\prime }}_{ij}=\bar{{\varepsilon^{\prime }}_{ji}}$:

(1) $$\varepsilon =\left(\begin{array}{ccc} \varepsilon_{xx} & \varepsilon_{xy}+{ig}_{z} & \varepsilon_{xz}-{ig}_{y}\\ \varepsilon_{xy}-{ig}_{z} & \varepsilon_{yy} & \varepsilon_{yz}+{ig}_{x}\\ \varepsilon_{xz}+{ig}_{y} & \varepsilon_{yz}-{ig}_{x} & \varepsilon_{zz} \end{array}\right)$$

where g is the gyration vector and $\varepsilon_{ij}\pm {ig}_{k}={\varepsilon^{\prime }}_{ij}$.

The refractive index n is related to the relative permittivity $\varepsilon_{r}$ through the following well-known equation:

(2) $$\varepsilon_{r}=Re\left(\varepsilon_{r}\right)+i\cdot Im(\varepsilon_{r})$$

where $Re\left(\varepsilon_{r}\right)=n^{2}-k^{2}$ and $Im\left(\varepsilon_{r}\right)=-2nk$.

In these relations, n and k are the real and the imaginary part of the refractive index, respectively, and $Re\left(\varepsilon_{r}\right)$ and $Im\left(\varepsilon_{r}\right)$ are the real and imaginary part of the relative permittivity $\varepsilon_{r}$, respectively.

Through the changes induced in the permittivity tensor, it is possible in principle to modulate the metasurface response.

Another source of modulation is the application of an electrical bias.

The application of an external electric field generates four types of effects, i.e., field effect in semiconductors, Pockels’ effect in ferroelectrics, molecules’ orientation in liquid crystals, and electro-thermal effect in phase change materials (PCMs).

The field effect consists of the modulation of the conductivity of an underlying semiconductor layer by the application of an electric field.

The relative permittivity of a semiconductor can be described using the Drude model. Thus, the real and imaginary parts of the permittivity can be described as a function of the plasma frequency, which is defined as the frequency at which the real part of the permittivity approaches nearly zero.

The plasma frequency is described in the Drude model by the following well-known equation [57]:

(3) $$\omega_{p}=\sqrt{\frac{Ne^{2}}{\varepsilon_{0}m}}$$

where N is the number of free carriers in the material, e is the electric charge of the electron, $\varepsilon_{0}$ is the dielectric permittivity of the vacuum, and m is the effective mass. Therefore, the application of an electrical bias introduces the modulation of the concentration of free carriers modifying the relative permittivity of the material.

The permittivity experienced by the incident electromagnetic wave can also be modified by another external electromagnetic field, exploiting the nonlinear electromagnetic response of the material. To account for nonlinear behavior of the material, the polarization is expressed as a series development of the electric field

(4) $$P=P^{\left(0\right)}+P^{\left(2\right)}+P^{\left(3\right)}+\ldots =\varepsilon_{0}\chi^{\left(1\right)}:E+\varepsilon_{0}\chi^{\left(2\right)}:EE+\varepsilon_{0}\chi^{\left(3\right)}:EEE+\ldots$$

where χ^(1)^ is the linear polarizability (second-order tensor) and χ^(*n*)^ is the *n*^th^-order nonlinear polarizability (*n*+1 order tensor). Owing to symmetry reasons, the second-order tensor is non-vanishing only in non-centrosymmetric materials (e.g., non-cubic crystals).

In particular, the Pockels’ effect is the linear electro-optics effect that can be induced in mediums (such as LiNbO_3_) without inversion symmetry like the ferroelectrics materials by an applied external electric bias.

In fact, the application of a direct current (DC) field perturbs the propagation of an incident electromagnetic wave at frequency ω by introducing a second-order polarization term ***P***^(2)^ = *ε_0_χ*^(2)^:***E***(*ω*)***E***(*DC*) acting as a modification of the linear permittivity tensor. Consequently, a birefringence is induced with the refractive index depending on the amplitude and polarization of the electric field.

The difference between the indices of refraction for light polarized parallel or perpendicular to the applied field is given by the following:

(5) $$\Delta n=r_{ij}n_{o}^{3}E$$

where $r_{ij}$ is the electro-optic coefficient, E is the magnitude of the DC electric field experienced by the crystal with the application of an applied voltage V, and $n_{o}$ is the ordinary refractive index.

For a strong external electric field in any kind of material, we can have the Kerr effect, which is closely related to the third component of the susceptibility $\chi^{\left(3\right)}$. The refraction index variation due to birefringence is described by the following relation:

(6) $$\Delta n=\lambda KE^{2}$$

where λ is the wavelength of the incident electromagnetic field, K is the Kerr constant, and E is magnitude of the DC electric field experienced by the crystal with the application of an applied voltage V.

In non-centrosymmetric materials, the Kerr effect is usually masked by the much stronger Pockels’ effect.

In the case of liquid crystal, the application of an electrical bias can induce anisotropy in an isotropic material or can change the orientation of the optical axis in an already anisotropic material. These phenomena are due to the alignment of the LC molecules, which arrange themselves parallel to the electric field, modifying the dielectric function of the material.

PCMs such as germanium antimony tellurium (GST), Ge_2_Sb_2_Se_4_Te (GSST), antimony sulfide family (Sb_2_S_3_, Sb_2_Se_3_), and VO_2_ have the interesting property of possessing a large contrast between the refractive index in the amorphous and crystalline state, which can be reversibly switched on the nanosecond scale or faster, mainly by variation of temperature, by current or voltage, but also by excitation with light [58]. Such electrically tunable optical properties have enabled multiple pioneering applications of reprogrammable metasurfaces [59].

Optical stimuli generally modulate the free-carrier density in an active material through photocarrier excitation. This changes the complex refractive index and thus the permittivity tensor of the material and eventually its optical properties. The most responsive materials for optical modulation of charge carrier density are the semiconductors.

Temperature is another external stimulus that allows the changing of the dielectric properties of a material such as BaTiO_3_. For solid-state matter, we can assume that $\frac{dn}{dT}$ depends on both the change in the refractive index with temperature at a constant density and the change in the refractive index with density at a constant temperature, as described by the following equation [60]:

(7) $$\frac{dn}{dT}={\left(\frac{dn}{dT}\right)}_{\rho }+{\left(\frac{dn(\rho )}{d\rho }\right)}_{T}\left(\frac{d\rho (T)}{dT}\right)$$

where *ρ*(*T*) is the temperature-dependent density. $\frac{dn}{dT}$ can assume negative or positive values on the basis of increasing or decreasing ρ(T) with temperature. Thus, the temperature modifies the real part of the material refraction index and, consequently, the diagonal values of the permittivity ε.

Temperature tuning is not only directly linked to density variation; in the case of nematic liquid crystals, the temperature is responsible for the switch from the anisotropic system to an isotropic state through the increase in entropy [61]. As a matter of fact, the nematic liquid crystals above a certain temperature threshold become isotropic.

Another example of thermal tuning is the phase change that occurs in materials such as VO_2_, in which a phase change was activated either purely by thermal heating or by electro-thermal heating [62,63].

As previously explained, this class of compounds can be considered as both electrically and purely thermally tuned active materials.

Furthermore, we can modulate the permittivity of a material with mechanical stress. In fact, under the action of mechanical stresses, the refractive index of isotropic or anisotropic media can change, manifesting either a new acquired optical anisotropy or a change in the previous anisotropy. Therefore, the internal stress could be generated through mechanical stress factors that destroy the isotropy and produce birefringence or generate a different anisotropy than the previous one by modifying the birefringence of the material.

**Figure 2:**
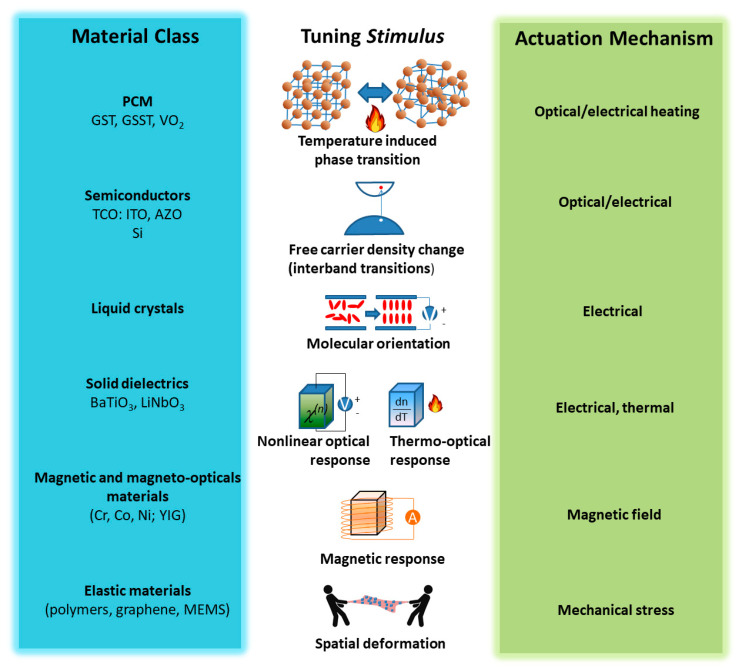
Schematic representation of the principal tuning methods with the class of related materials.

Figure 2 summarizes in a schematic way the material classes and the respective tuning mechanisms to which they are sensitive.

### 2.1. Electrical Bias

Tunable metalenses, through electrical bias, can be realized by applying an external electrical stimulus (i.e., current or voltage) on active materials, such as LCs [49,64,65,66,67,68], graphene [69,70,71,72,73,74], semiconductors [75,76,77,78], or PCMs [79]. The modulation of the refractive index of a medium, directly in contact with the nanostructures, is one of the most widely used methods to realize tunable metasurfaces. Among the various tunable substrates, LCs are certainly the ones with wider uses.

Komar et al. [64] demonstrated electrical tuning of the transmission spectrum of a Mie-resonant dielectric metasurface consisting of silicon nanodisks embedded into anisotropic nematic LCs. They achieved experimental transmission modulations of up to 75% at λ = 1550 nm and a phase change of up to approximately π by an applied alternate current (AC) bias voltage of 70 V at 1 kHz. This type of metasurface could have a broad spectrum of applications including dynamic holography, tunable imaging, optical signal modulation, and active beam steering.

**Figure 3:**
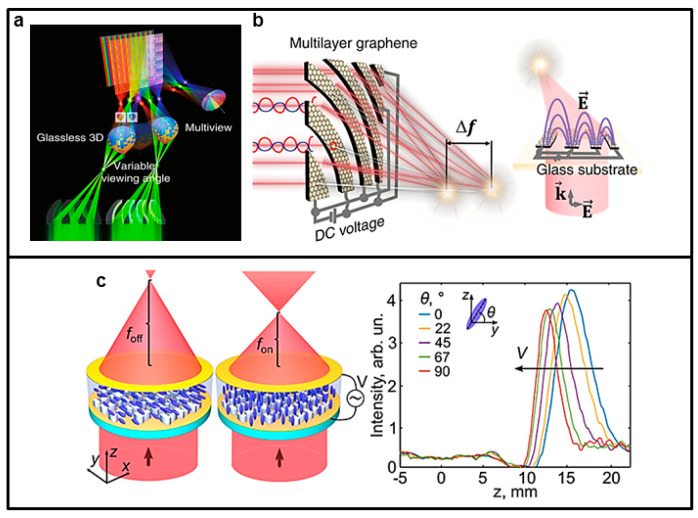
Examples of two electrically tunable metasurfaces. Upper panel (**a**,**b**): (**a**) Ultrathin square subpixel lens (USSL) capable of electrically tunable focusing (ETF) in a display. Multi-focus is achieved using an ultrathin square subpixel lens, thanks to which glassless 3D and multiview displays are implemented. The characteristics of the electrically tunable focusing allow for a variable viewing angle. (**b**) Focusing through the multilayer graphene arc ribbon pattern. **a**,**b** were adapted from [69] under the terms Creative Commons CC BY licence. Lower panel (**c**): Ultrathin varifocal metalens encapsulated in an electrically biased liquid crystal cell: the focal length continuously varies from f_off_ (no bias) to f_on_ (voltage on). Alongside, intensity vs. the optical axis z for five values of angle θ. **c** was adapted with permission from [65]. Copyright 2021 American Chemical Society.

In the field of optical applications, compact varifocal lenses have always been of great interest and essential for various imaging and machine vision technologies. As a matter of fact, varifocal lenses are extremely important for focusing and imaging in three dimensions. In traditional optics, the modification of the focal length of a lens (e.g., a photographic zoom lens made of high-precision glass optics) is usually achieved by mechanically moving one or more optical elements of the lens, which results in heavy and bulky systems. Thanks to metasurfaces, it is possible to achieve focal length control through a single element in a compact and lightweight manner. In this regard, in a recent study, Bosch et al. [65] experimentally demonstrated an ultrathin LC-based metalens operating in the visible spectral region whose focal length can be adjusted between 9 mm and 4.5 mm when a low voltage bias of 9.8 V is applied (See Figure 3c). This kind of metasurface is composed of a nanostructured array of amorphous silicon encapsulated in an LC cell that is sandwiched between two biasing indium tin oxide electrodes. The most distinctive feature is that the varifocal lensing is reversible, allowing switching between two discrete focal lengths. 

**Figure 4:**
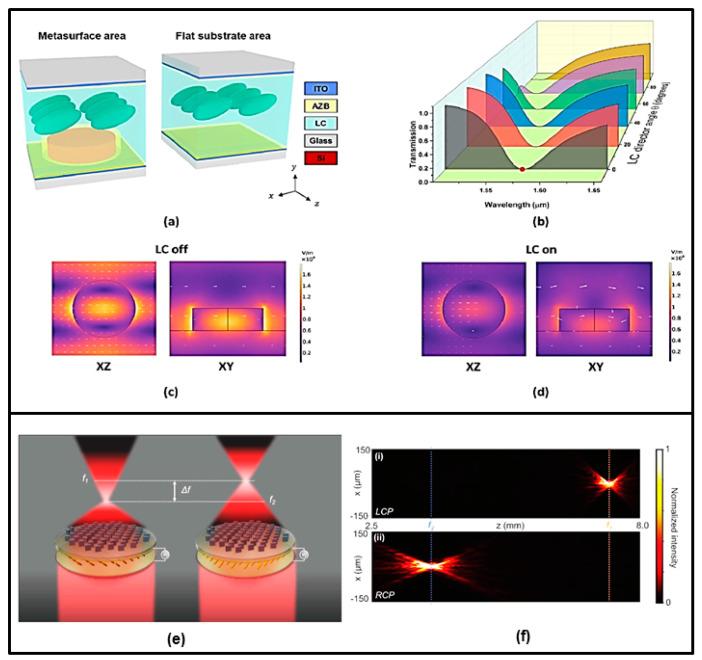
Examples of two LC-based electrically tunable metasurfaces. Upper panel (**a**–**d**): (**a**) Structure of LCAM unit cell illuminated with linearly polarized light, along the Z direction and at normal incidence. (**b**) Simulated transmission spectrum of the LCAM at various LC director angles θ from 0° to 90°. The electric field distribution at 1575 nm in the XY plane and XZ plane when LC is off (**c**) or on (**d**). **a**–**d** were adapted from [49] under the terms Creative Commons CC BY licence. Lower panel (**e**,**f**): (**e**) Schematics of the bifocal metalens in which, by applying an external bias to the LC cell, the orientation of the LCs causes a change in the emerging light between RCP and LCP, which in turn produces a change in focal length from f1 (7.5 mm) to f2 (3.7 mm). (**f**) Optical field intensity profile along the x–z plane for LCP and RCP incident light. (**e**,**f**) were adapted from [67] under the terms Creative Commons CC BY licence.

Another work on varifocal lensing is that of Badloe et al. [67]. The authors designed and realized an electrically tunable diffraction limited focusing metalens operating at visible wavelengths. The metalens is composed of hydrogenated amorphous silicon rectangular nanorods and incorporates an LC cell to modulate the effective local refractive index around the metalens, with geometry parameters optimized for working at λ = 633 nm. Specifically, the application of an electrical bias to the LC cell induces a modulation of the linearly polarized incident light, causing the emerging light to switch between right circular polarization (RCP) and left circular polarization (LCP), and determining the focal length to shift between 7.5 and 3.7 mm with focusing efficiencies of 43.5% and 44.0%, respectively. In addition, active focus imaging on a millisecond time scale using bifocal lenses at a wavelength of 633 nm has also been experimentally demonstrated. Some artifacts in the images were attributed by the authors to an imperfect conversion between light LCP and RCP and manufacturing defects (see Figure 4e,f). 

The study by Xin Chang et al. [49] demonstrated that LC-based active metasurfaces (LCAMs) provide wide amplitude modulation with fast device communication, giving a practical option for optical switches in telecommunication networks (see Figure 4a–d). The metasurface operates at λ = 1550 nm and is composed of silicon nanodisks placed on top of an indium tin oxide (ITO)-coated borosilicate glass substrate. The nanodisks are arranged in a periodical square lattice. The cell is filled with nematic LC and another ITO-coated borosilicate glass is used as the ceiling of the LCAM cell. A shift in the spectral position of the resonance of as large as 49 nm was obtained in the C-band with applied voltage variation in the range of 0–10 V. A maximum modulation depth of 94% in the transmission amplitude was recorded at 1530 nm and a submillisecond speed of the LCAM operation was demonstrated at 1525 nm and 60 °C. LC technologies, as well as the scalability of silicon nanostructures’ production, are very mature; for this reason, the LCAM proposed by Xin Chang et al. [49] shows great application perspectives.

Another effective material for electrically tunable matasurfaces is graphene. Indeed, graphene is an ideal platform for optoelectronics and optics because its sp^2^-hybridized orbitals can allow electrical control of the optical response in suitably designed systems. Sehong Park et al. [69] developed a graphene-based ultrathin square subpixel lens (USSL) capable of electrically tunable focusing (ETF); see Figure 3a,b. The tunable focusing capability was developed to realize a multifunctional autostereoscopic display. A focusing efficiency of over 60% was achieved at a visible wavelength of 405 nm with a lens thickness <13 nm. A 19.42% change in focal length with a 9% increase in transmission was demonstrated experimentally with a DC voltage bias. An important point is that the performance of this metasurface is competitive with the typical mechanical refractive lens.

Zhao Kun Zhang et al. [71] demonstrated a dynamic focusing metalens in the THz band based on a hybrid structure of simple metal strip arrays and unstructured monolayer graphene. The metasurface consists of an unstructured graphene layer decorated with Au antennas. The response of the graphene is modulated by injecting free carriers by means of an external electric field. Single-layer graphene sits on a dielectric layer of SiO_2_ supported by a thick Au film. Focusing can be shifted by ∼3λ and the maximum focusing efficiency reaches 61.62%. These results indicate a large application potential in the terahertz band.

The realization of highly efficient programmable electro-optics devices such as tunable electro-optic modulators, near-infrared optical switches, and so on is currently a technological challenge. This challenge can be tackled through electrically tunable metasurfaces, as shown by the work of Yankai Chen et al. [72]. In this paper, they designed a hybrid graphene–silicon metasurface that supports toroidal Fano resonances and a high-quality factor electrically tuned in the near-infrared band with voltage ranging from 0 to 2 V. Specifically, the nanostructures consist of an array of silicon nanocylinders cut by a notch at the margin of the dielectric cylinder to break their symmetry.

The Fano parameters are tuned in the range −0.32 to −0.88 by applying the bias voltage to a single-layer graphene obtaining a ∼4 nm blueshift of resonant wavelength with a maximum change in the transmission spectrum peak of up to 30%. Another interesting point is that the production methods of single-layer graphene are in an advanced state, and the realization is highly feasible.

To date, there are technological challenges in the realization of reconfigurable terahertz devices with high tunability. As a matter of fact, the carrier frequencies of wireless systems have shifted to millimeter wave (mmW) and terahertz (THz) bands to enable high data rate wireless communications and super resolution imaging. The lack of materials and technological solutions capable of working in these spectral regions has slowed the development of THz wireless communication devices. On this subject, Andrew D. Squires et al. [73] specially designed and developed a graphene bilayer metamaterial structure with highly tunable THz frequency selective absorption functionality. Through their work, they demonstrated the superiority of this dual layer approach designed to operate in the 0.1–1 THz communication window. The metasurface consists of a 2 cm × 2 cm graphene/gold bilayer, i.e., a thin film of graphene transferred onto a 200 nm gold film. This graphene/gold bilayer features a multitude of 450-µm-sized Jerusalem-cross-slots arranged in an ordered manner. The metasurface is implemented on a flexible glass-reinforced PTFE substrate (Rogers5880 Duroid) of 254 µm thick and, on the back of the substrate, we have another 200 nm thick gold film. Such a metasurface changes its reflectivity of more than 16 dB with a bias voltage of only 6 V while maintaining a high reference Q factor (up to 19); moreover, it shows a broadband tuning of more than 95% with the same low bias at 6 V. Hence, in this work, the increased effectiveness of the gold–graphene bilayer approach is demonstrated, and it is displayed that it outperforms the performance of THz metasurfaces with a single layer of graphene, which generally show a lower Q factor. Furthermore, this work has a great impact in emerging THz fields such as THz wireless communications.

Another class of materials that are very sensitive to voltage or current application are semiconductors. A very interesting aspect is that the refractive indices of semiconductors can be tuned by altering their carrier densities and effective masses. This can be modeled using the Drude model (Equation (3)), which links the plasma frequency and, therefore, the refractive index to the carrier density and to their effective mass. For example, silicon, one of the most widely used semiconductors, can be transformed from insulator to metal in the terahertz (THz) spectral range with optically injected carriers. The same happens in compounds such as ITO [77] and AZO [78], where the transition from pure semiconductors to plasmonic material is obtained by chemical doping.

As far as semiconductors are concerned, a very interesting work is that of Forouzmand et al. [76]. They proposed a tunable semiconductor-based metasurface dependent on electro-optical modulation of an array of silicon nanogratings sandwiched between two distributed Bragg reflectors. The refractive index of silicon nanorods is electrically modulated by the injection of holes and electrons using a P-I-N junction configuration along each nanorod. The entire geometry was refined in order to excite two spectrally close Fabry–Pérot and guided-mode resonances. This allows a flat-top transmission amplitude and steep transmission-phase spectra around the telecommunication wavelength (1.55 µm). In this project, a variation in the refractive index of ∆n_Si_ = 0.01 was achieved that allows a phase modulation of 215°. In the whole phase coverage, the transmission amplitude is greater than 0.6, with an average level of 0.83. This active design has the best trade-off between phase agility and transmission amplitude compared with previously proposed phase modulator designs in the NIR region.

Another kind of active material responsive to an electrical stimulus are PCMs. PCMs are promising materials for reconfigurable optical functionalities thanks to reliable and repeatable electrically induced and temperature induced switching of their optical and electrical properties.

Zhang et al. [79] have realized reconfigurable metasurfaces via electrical bias, composed of Ge_2_Se_2_Sb_4_Te (GSST) nanostructures. GSST is transparent in the near-infrared region, allowing for engineering of thicker metasurfaces. They demonstrated an on-chip electrical switching platform enabling quasi-continuously tunable active metasurfaces with recording of half-octave spectral tuning from 1190 nm to 1680 nm. Thus, they proved to attain ultra-broadband resonance tuning. More precisely, this kind of metasurface consists of square nanostructured PCM layer (GSST) with a total thickness of 250 nm and unit cell periodicity of 800 nm, unlike devices reported by others authors, where the PCM layer is <100 nm. The authors also demonstrated, on a very similar platform to the previous one, to be able to reach a large absolute optical reflectance contrast of 40% at a wavelength of 1.49 μm and a relative reflectance modulation up to 400% at a wavelength of 1.43 μm. This platform is composed of a tunable metasurface consisting of a periodic array of square-shaped identical GSST meta-atoms with size of 620 nm and height of 230 nm in a unit cell with 800 nm sides. Furthermore, they demonstrated and realized a polarization-insensitive phase-gradient metasurface. This metasurface realized dynamic optical beam steering, operating at 1.55 μm with a normally incident electromagnetic field. It is composed of an asymmetric unit cell consisting of two GSST disks of a different radius. This asymmetry allows the metasurface to work as a deflector of the optical beam in the amorphous state and, mainly, as a reflector in the crystalline state of GSST.

### 2.2. Magnetic Field

Tunable magnetic structures represent a cutting-edge research field because an instantaneous response to magnetic stimuli without any kind of contact makes this type of metasurface extremely attractive. Of great interest are metasurfaces composed of magneto-optical (MO) materials that can increase the Faraday rotation and, in recent years, several research works have investigated this peculiar aspect.

On this subject, a very interesting work is that of Aristi Christofi et al. [80]. They proposed a new class of Fano-resonant all-dielectric metasurfaces operating in the NIR spectral range (1360 nm–1490 nm) that are able to simultaneously obtain a large Faraday rotation enhancement along with almost 100% transmittance, owing to electromagnetically induced transparency of the metasurface. The structure under study is a metasurface of yttrium iron garnet disks arranged into a square array embedded into a silica matrix. It is very important to outline that these results were obtained for the first time in an all-dielectric nanostructure. Thus, this typology of metasurfaces offers high rotation angle values with application outlets for active optical devices tunable by an external magnetic field in subwavelength thickness structures.

Fei Fan et al. [81] proposed a tunable metasurface for dynamic THz polarization manipulations, induced intrinsic chirality, and active spin state manipulation through an external magnetic field that transversely magnetized an indium antimonide (InSb) substrate. The structure is composed of a nanostructured triple rotating split metallic ring array on a substrate of InSb. Compared with previous works describing active chiral metasurfaces [82,83], in this work, the same tunable chirality is achieved through a magneto-optical metasurface with only one layer of metallic microstructure. This design avoids the need for more complex structures such as in the case of graphene- or VO_2_-based metasurfaces. Thus, we see an interesting example where magneto-optical material is employed. These materials present important advantages in the magnetic field control of the photonic chiral spin states and in the intrinsic transmission. At the applications level, the ability to manipulate the photonic spin state and optical chirality opens horizons to the enhancement of polarization sensitive imaging techniques, chiral spectroscopy, and multi-channel communication.

An active field of research addresses the materials responsive to a magnetic field modulation. A very interesting typology in this regard is the family of magnetoplasmonic materials. Such materials exhibit plasmonic and ferromagnetic behavior at the same time. Another approach is to nanostructure metasurfaces by combining plasmonic materials such as gold and ferromagnetic materials such as nickel. This was studied by Irina Zubritskaya et al. [84], who simulated and realized a plasmon surface with chiroptic response controlled by an external magnetic field in the visible and near-infrared spectral ranges. The nanostructure is composed of repeating trimers of three near-field coupled nanometric disks with diameters close to 100 nm and height of 30 nm, one of which consists of nickel (a ferromagnetic material) and the other two of gold (a noble metal). The magnetic-field-induced modulation of the chiroptic far-field response with this surface is regulated up to 150% by the external magnetic field in the visible and near-infrared spectral ranges. This paves the way for possible optical applications whose response can be tuned in real time.

**Figure 5:**
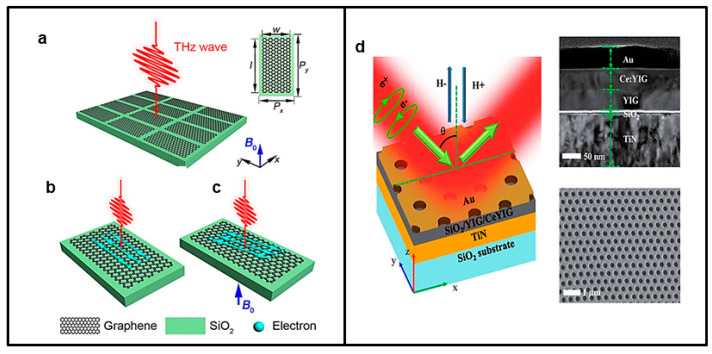
Examples of two magnetically tunable metasurfaces. Left panel (**a**–**c**): (**a**) Graphene-based terahertz metasurface with structural parameters: P_x_ = 250 μm, P_y_ = 500 μm, l = 450 μm, w = 210 μm, and h = 50 μm (SiO_2_ substrate thickness). Oscillation of the electrons in the unit cell (**b**) without or (**c**) with the external magnetic field. Figure 5a–c were adapted from [86] under the terms Creative Commons CC BY licence. Right panel (**d**): Magnetoplasmonic metasurface schematics and operation mechanism. An out-of-plane magnetic field modulates the far-field extrinsic chirality under oblique incidence conditions. Cross-sectional image of the device characterized by TEM and surface morphology of the device measured by SEM. Figure 5d has been adapted with permission from [85]. Copyright 2020 American Chemical Society.

In another work on chiroptic applications, Jun Qin et al. [85] reported the switching of extrinsic optical chirality in magneto-optical metasurfaces by applied magnetic fields from −0.6 ± 0.2 to +1.9 ± 0.1 at a wavelength of 950 nm under oblique incidence conditions. The structure, shown in Figure 5d, is composed of three layers: an SiO_2_ substrate, a TiN layer, a Ce:YIG layer, and finally a hexagonal periodic nanohole structure in a Au thin layer. Ce:YIG causes low optical loss and a strong magneto-optic effect, which allows obtaining giant and continuous far-field circular dichroism (CD) modulation by magnetic fields. This study has opened a broad perspective on active chiral nanophotonic devices, where optical chirality can be controlled by an external stimulus for integrated polarization control, sensing, and display applications.

An external magnetic field could also be employed to modulate the transmittance of the incident wave of metasurfaces. In this regard, Yafeng Lu et al. [86] proposed a tunable graphene metasurface on top of a SiO_2_ substrate whose transmittance in the THz is regulated by an external magnetic field. The metasurface and its operation principle are presented in Figure 5a–c. Their results provide a new future perspective for the design of graphene-based dynamically and magnetically tunable THz metasurfaces.

Armelles et al. [87] proposed a metasurface composed of arrays of aligned rods and slits fabricated from giant magnetoresistance Ni_81_Fe_19_/Au multilayers operating in the mid- and far-IR range. Thanks to the metasurface they designed, they managed to modulate the reflection and transmission spectra in the far field. Instead, in the near field, they achieved a fivefold amplification of the electric and magnetic fields via magnetic tunability. Their results led to the exploration of the near field behavior, in addition to the properties of the far field. This can have a great impact on applications where the local field properties are more significant.

Karmakar et al. [88] designed and experimentally demonstrated optically thin terahertz metasurfaces operating in the sub-skin depth regime, where both dipole and Fano resonances are dynamically tuned by an external magnetic field in the range 0–30 mT. The transmittance range of these magnetically tuned metasurfaces is 0.3 to 1.4 THz. Metasurfaces have an asymmetrical structure; in particular, they are composed of a superlattice arrangement of a magnetic layer such as Ni on a non-magnetic layer such as aluminum. A silicon wafer with a thickness of about 500 μm was used as substrate. Thin layers of aluminum (Al) and nickel (Ni) of 10 nm are periodically deposited on top of this wafer. Both four-layer and eight-layer Al/Ni structures were created. The dimensions of the metasurface are 1 cm × 1 cm. The results obtained by Karmakar et al. [88] metasurfaces’ behavior are due to spin-dependent terahertz magneto transport phenomena in metals and may stimulate the paradigm for on-chip spin-based photonics technology.

Liquid crystals are highly tunable materials with an external electric field, but they can also be tuned magnetically.

Yana V. Izdebskaya et al. [89] demonstrated magnetic field tuning of liquid crystal dielectric metasurfaces. The structure is composed of an SiO_2_ substrate, a zigzag nanostructured array of elliptical silicon cylinders integrated in a nematic liquid crystal (NLC) cell. The orientation of the NLC molecules is controlled by an external magnetic field generated by a permanent neodymium magnet mounted at a distance of 4 mm from the metasurface. The change in the orientation of the external magnetic field leads to different orientations of the NLC molecules and, therefore, to a variation in the refractive index and consequent transmission modulations of up to 42% and a maximum dynamic tuning range for resonances of 37 nm at infrared wavelengths of around 1500 nm.

### 2.3. Temperature

A further way to tune a metasurface is by heating or cooling, taking advantage of the thermo-optic effect. In silicon, the thermo-optic effect has been used for a long time to obtain shifters and tune resonant cavities [90]. In a recent paper [51], Rahmani et al. demonstrated that the thermo-optic effect can be used to tune nanoscale resonators based on crystalline silicon nanodisks on sapphire substrate arranged in a two-dimensional metasurface, operating in the spectral window between 650 and 1500 nm. The metasurface showed sharp resonances under the excitation of magnetic dipole and electric quadrupole modes. Heating and cooling of the metasurface allow obtaining drastic but reversible changes in the forward scattering (by heating) and backward scattering (by cooling) from the metasurface at specific wavelengths in the temperature range of 20–300 °C. The authors tested three different metasurfaces with nanodisks having diameters of 170 nm, 470 nm, and 770 nm, respectively. All of the resonator’s configurations experienced a similar redshift of about 30 nm, in agreement with theoretical simulations. This type of tunable metasurface can be implemented for the development of flat optical devices such as metalenses and metaholograms.

Tian et al. [91] experimentally realized a reconfigurable dielectric perovskite (BA_2_PbI_4_) metasurface whose polarization-dependent optical response and dynamic control of structural color and emission wavelength can be tailored by temperature modulation.

A BA_2_PbI_4_ perovskite sub-micrometer nanograting metasurface was obtained by nanoimprint lithography on a quartz substrate. The reflected color is dynamically controlled in the range of 400–800 nm through the switching of crystallographic phases of the perovskite by varying the temperature between 293 and 240 K. Possible applications of this metasuface can be suitable for light-emitting devices, displays, and spatial-light modulators.

**Figure 6:**
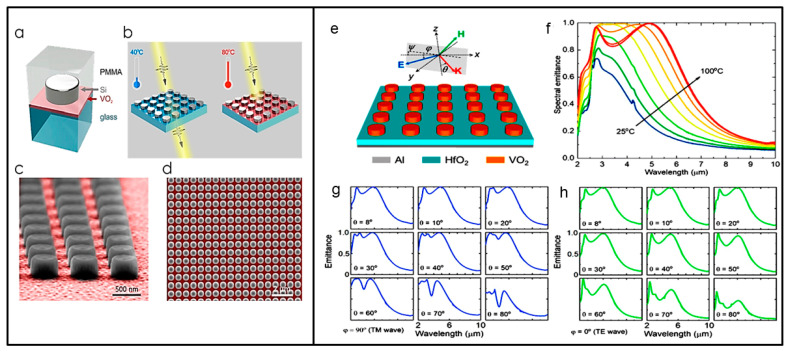
Example of two temperature-tunable metasurfaces exploiting the insulator to metal transition of VO_2_. Left panel (**a**–**d**): Design: silicon disk (height: 240 nm, disk radius: 255 nm) embedded into PMMA (500 nm) surrounding a thin VO_2_ layer (25 nm). (**b**) Tunable extinction with temperature variation. (**c**,**d**) Electron microscope images of the fabricated Si–VO_2_ metasurface prior to the PMMA coating. Adapted with permission from [92]. Copyright 2021 American Chemical Society. Right panel (**e–h**): (**e**) Schematic of the structure and directional measurement. (**f**) FTIR measurement at different temperatures (from 25 to 100 °C) with unpolarized light and near-normal incidence. (**g**) Spectral emittance of transverse magnetic and (**h**) transverse electric waves. Adapted with permission from [93]. Copyright 2020 American Chemical Society.

Tripathi et al. [92] demonstrated the reversible temperature tuning of Mie-resonant silicon-based metasurfaces, tunable via the insulator-to-metal transition of a thin layer of VO_2_ and operating at 1.55 µm. The metasurfaces tested are based on silicon cylinders embedded in PMMA on a VO_2_ layer of 25 nm (see Figure 6, Left Panel). The authors experimentally demonstrated two ways of exploiting the VO_2_ insulator to metal transition to obtain metasurface tunability, in transmission through the tuning of its refractive index at a temperature of 40 °C, and in absorption by tuning its extinction coefficient at a temperature of 80 °C, at which a near-perfect absorber performance is reached. The reversible temperature tuning mechanism is based on the redistribution of the electromagnetic energy between different multipoles of the dielectric disks as well as the large modification of the absorption loss with temperature changes.

Another temperature-tunable metastructure based on VO_2_ microdisks on a bylayer of aluminuum (Al) and afnium oxide (HfO_2_) working at 5 µm was proposed by Long and coworkers [93]; see Figure 6 (Right Panel). The metastructure infrared radiative properties were comprehensively characterized at different wavelengths, temperatures, incident angles, and polarization states. The metasurface experienced an enhancement in infrared emissivity by more than 180% upon phase change with temperature from 40 °C (ε = 0.08) to 80 °C (ε = 0.23) and finally reached 0.33 at 160 °C. Temperature-dependent infrared spectroscopy clearly showed that the spectral emittance at wavelengths from 2 to 6 μm is significantly enhanced when heated beyond its phase transition temperature, where the magnetic polariton is excited with metallic VO_2_ (see Figure 6, Right Panel).

Linyang et al. proposed a tunable THz metamaterial based on a Germanium antimony telluride, Ge_2_Sb_2_Te_5_ (GST), microdisk on a silicon substrate [94]. As a matter of fact, GST is thus far the most used and studied phase change material for electronic memory thanks to its suitable crystallization temperature, amorphous to crystalline resistance contrast, and stability of the amorphous phase. In the metasurface proposed in [94], three resonance modes are excited to obtain two absorption peaks (P1 at 1.98 THz and P2 at 5.88 THz) and one absorption valley V1 in the region at 6.32 THz. P1 and P2 are enhanced and shifted to lower frequencies with the increasing diameter D, while, when the microdisk thicknesses increases, only P1 is enhanced and shifted to a higher frequency. Resonance frequencies of P1, P2, and V1 can be tuned by an increase in temperature. In particular, the resonance position of absorption peak P2 can be covered by absorption valley V1 as a function of the specific crystallinity condition, thus determining the on or off state by changing the temperature. For these properties, this metamaterial can be useful in sensing, communication, and light modulation applications.

Another kind of metasurface operating in the same THz window and based on VO_2_ was proposed by Zhang et al. [95]. This structure consists of three-layer patterns separated by a dielectric substrate (lossy polyimide). The metal gold is the first layer at the bottom and is composed of different annular structures. The second layer is situated in the middle and features patterns made of VO_2_; the third VO_2_ layer consists of two octagonal ring-shaped resonators. It can be tuned by controlling, using the temperature, the phase transition of VO_2_. As a matter of fact, the metasurface shows a polarization-insensitive ultra-broadband absorption from 4.72 THz to 9.88 THz with absorptivity exceeding 90% when the temperature reaches 350 K (above the phase change temperature of VO_2_) and a single-band absorption when the VO_2_ resonators are in the insulator phase (340 K). The potential applications of this thermally switchable and tunable metasurface in the THz regime are radar stealth and thermal detection.

In a recent paper [96], a metasurface based on barium titanate (BaTiO_3_) topped by a strip coupled to two U-shaped resonators made of aluminium metal on a MgO substrate, fabricated with standard photolithography, was characterized using THz time domain spectroscopy. The transmission through the samples was measured at different temperatures in the spectral window of 0.4–0.9 THz. By increasing the temperature from 25 °C to 100 °C, the resonance dips undergo a red shift of ∼27 GHz with amplitude modulations. The authors attributed the red shifts and amplitude modulations of the THz spectra to the temperature-dependent dielectric property changes of the ferroelectric BaTiO_3_ thin film. The dynamically tunable electromagnetically induced transparency effects demonstrated in this work can be applied for the development of tunable devices such as optical switches and filters.

### 2.4. Mechanical Stressors

Using mechanical deformation, the optical response of a metasurface can be tuned to obtain programmable optical responses such as tunable focusing, plasmonic resonances, and dynamic colour switching [97,98,99]. The application of mechanical stimuli to deformable substrates induces a change in the periodicity and/or distance between adjacent elements without altering the shape of meta-atoms. The shift induced in the unit cell periodicity gives rise to a change in the metasurface optical response. Micro-electro-mechanical systems (MEMS) technology offers the possibility for tuning metasurface electromagnetic properties by structural reconfiguration [99,100,101,102,103,104].

MEMS actuators can be electrostatic, electrothermal, and piezoelectric. Electrostatic MEMS employ oppositely charged electrodes, i.e., a stationary one and a so-called membrane separated by a tunable distance. It offers a large range of motion, satisfactory speeds, and low power consumption.

A very recent paper proposed a new way of tuning graphene-based THz metasurfaces using an electromechanical method [105]. The metasurface consists of a free-standing graphene layer supported by an oxide grating on a gold substrate. Electromechanically, the metasurface acts as a variable capacitor, while electromagnetically, it is a frequency-dependent tunable absorber. Any potential difference between the graphene layer and the gold substrate causes a deflection in the free-standing regions of the graphene layer, which in turn causes a shift in the resonance wavelength of the metasurface. The authors demonstrated that the maximum bandwidth of operation of the modulator is 353 kHz. At an operating frequency of 5.49 THz, they obtained an extinction ratio of 4.67 dB for a drive voltage of 20 V. This tunable THz metasurface finds application in optical MEMS-based sensors, actuators, beam steering, and others.

Thrane and coworkers [106] studied and realized a platform combining MEMS and gap-surface plasmon (GSP) metasurfaces operating at 800 nm. The metasurfaces studied in this paper are based on a gold MEMS mirror and a glass substrate with gold brick-shaped nanostructures (with site lengths and thickness of 50 nm), where the air gap between the nanostructures and mirror can be controlled accurately by MEMS in order to obtain high modulation efficiency, broadband operation, and fast response. They compared tunable metasurfaces operating in GSP and Fabry–Pérot (FP) regions by investigating polarization-independent blazed gratings. The calculated peak efficiency is ∼75% in both cases (∼40% in measurements), while the operation bandwidth is found to be larger when operating in the GSP region. Moreover, their results showed that coupling between neighboring unit cells increases for larger air gaps, resulting in deteriorated efficiency. This platform could be implemented for diverse applications in miniaturized adaptive optical systems.

Holsteen and colleagues realized a fast MEMS tunable metasurface based on suspended silicon antenna arrays that offers a phase control from 0 to 2π and large amplitude modulation of the scattered waves [107]. In particular, the device is composed of a periodic nanowires array with a thickness of 100 nm and widths linearly graded from 80 to 160 nm. It can continuously steer 600 nm light with an electrical bias of a few volts, which can also be used to select and mix certain colors, as well as for light focusing.

Another approach to obtain mechanically tunable metasurfaces is the integration of meta-atoms in deformable polymeric substrates such as polydimethylsiloxane (PDMS), polyamide (PI), polyethylene terephthalate (PET), polyethylene naphthalate (PEN), poly(methyl methacrylate) (PMMA), low-density polyethylene (LDPE), silicone (SI), and cyclic olefin copolymer (COC) [97,108]. Generally speaking, the deformable polymeric material must have a low elastic modulus to allow easy and repeatable deformations via stretching or bending, a low refractive index to minimize reflection loss, and a low absorption coefficient to enhance the transmission and propagation of electromagnetic waves.

**Figure 7:**
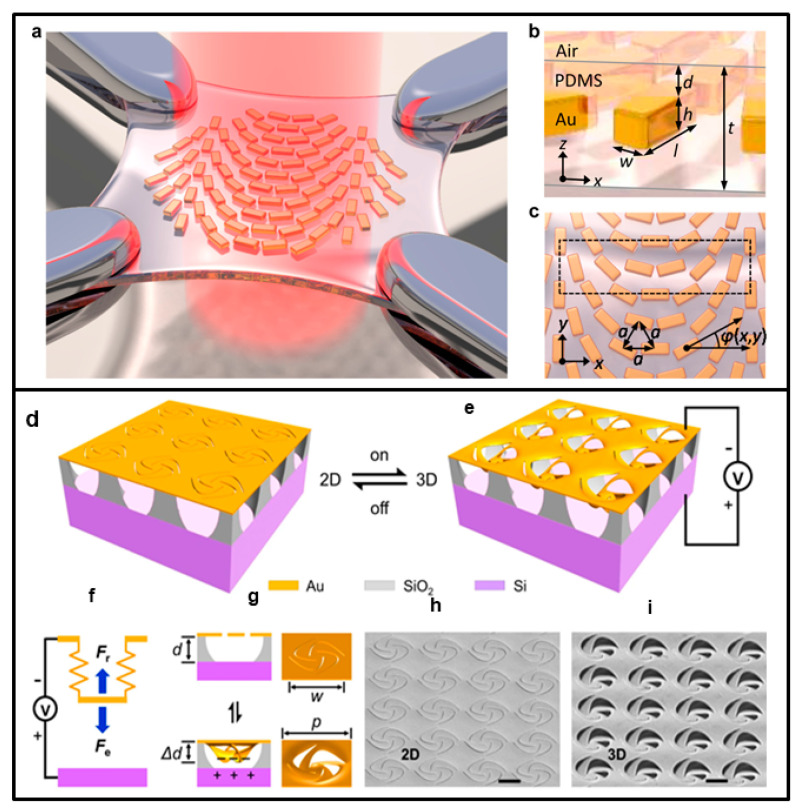
Example of two mechanically tunable metasurfaces. Upper panel (**a**,**b**): (**a**) Illustrations of a metasurface on stretched PDMS. (**b**) Nanorods’ dimensions: l = 240 nm, w = 100 nm, h = 70 nm, and d = 200 nm, respectively. (**c**) Orientation of Au nanorods on the polymer. Adapted with permission from [109]. Copyright 2016 American Chemical Society. Lower panel (**d**,**e**): (**d**) Illustration of a 2D pinwheel array and its transformation to a 3D state (**e**) under attractive electrostatic forces when the voltage is on. Each gold pinwheel is locally suspended by four SiO_2_ supporters. (**f**) Electromechanical model of the reconfigurable nano-kirigami, in which the displacement of the suspended nanostructure is controlled by the downward electrostatic force (Fe) and upward mechanical restoring force (Fr). (**g**) Front–view and side–view plots of (top) 2D and (bottom) calculated 3D deformed pinwheel. (**h**,**i**) Side–view SEM images of the 2D pinwheels and deformed 3D pinwheels after applying a DC voltage of V = 65 V. Adapted from [117] under the terms of Creative Commons CC BY licence.

A mechanically reconfigurable metasurface based on Au nanorod array (100 nm in length) on a stretchable PDMS substrate was realized by Ho-Seok Ee and colleagues [109]; its structure is reported in Figure 7a–c. The wavefront can be continuously tuned in the visible frequency range by changing the lattice constant; moreover, the refraction angle of incident visible light at 632.8 nm can be modulated from 11.4° to 14.9° by stretching the substrate. The authors also realized an ultrathin flat 1.7× zoom lens whose focal length can continuously be changed from 150 to 250 μm by stretching the substrate. This kind of reconfigurable metasurface can find application in information technology, biomedical sciences, integrated optics, optical communications, wearable consumer electronics, and so on.

A highly tunable dielectric metasurface based on subwavelength-thick silicon nano-posts encapsulated in a thin transparent layer of PDMS and operating at 915 nm was realized by Kamali and coworkers [110]. This metasurface can be used to realize a tunable metalens. Through the application of radial strain, this metasurface can be tuned, allowing to vary the focal distance from 600 μm to 1400 μm, maintaining a diffraction-limited focus and a focusing efficiency above 50%. This device can be employed for ultra-slim, multi-functional, and tunable optical applications.

Another class of mechanically tunable metasurface is represented by origami- and/or kirigami-inspired configurations. This kind of structure can be obtained by means of folding and cutting two-dimensional thin-film materials so as to transform them into complex 3D structures featuring unique and programmable mechanical properties [111,112,113]. An origami structure is folded from the initial planar state into a compact configuration, while a kirigami structure is stretched into an expanded configuration [112,114,115].

Han and colleagues demonstrated an electromechanically reprogrammable nano-kirigami metasurface for independent manipulation of pixels at visible wavelengths through mechanical deformation of the gold nanostructures [116]. Through the somministration of electrostatic forces between the top suspended gold nano-spirals and bottom silicon substrate, out-of-plane deformation is induced, allowing the control of each pixel and its associated phase retardation. Such a kirigami-ispired metasurface enables diverse dynamic optical and photonic reconfiguration in the visible wavelength range, and can be applied to other configuration designs, material platforms, and wavelength regions.

A similar approach is reported in the paper of Chen and coworkers [117], which describes the functioning of an on-chip and electromechanically reconfigurable nano-kirigami with optical reversible functionalities. The structure and functioning scheme of this metasurface are shown in Figure 7d–i. The nano-electromechanical system is also built in this case on an Au/SiO_2_/Si chip, where the electrostatic forces between the top suspended gold patterns and bottom silicon substrate actuate the 3D nano-kirigami transformations. The reflectance exhibited huge changes in the range of 400–1100 nm with the increase in direct current voltage, owing to the 3D vertical structure deformation. The reflectance can be modulated with current somministration and the process is reversible. The proposed nano-kirigami could be applied to a wide variety of material platforms for reconfigurable optical circuits and networks.

### 2.5. Optical Modulation

Metamaterials can change their optical properties in response to light interaction owing to the induction of photocarrier dynamics, which take place on very fast time scales, within femtoseconds or picoseconds [118,119,120].

**Figure 8:**
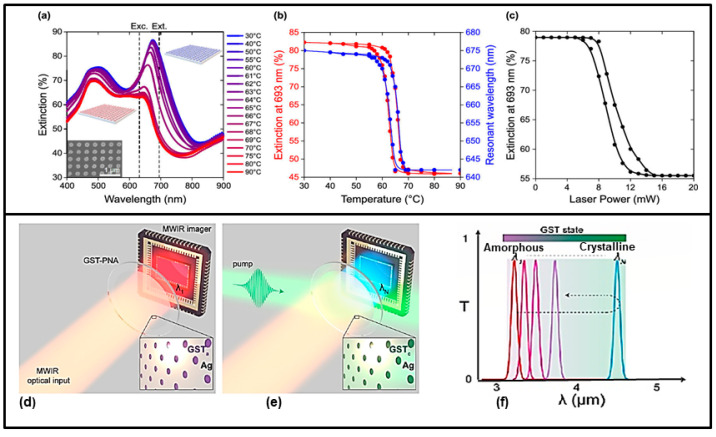
Example of two optically tunable metasurfaces. Upper panel (**a**–**c**): (**a**) Experimental extinction spectra of a VO_2_ nanodisk array during the heating cycle, which drives VO_2_ through its phase transition. The inset is a SEM micrograph of the array. The dashed vertical lines label the excitation (Exc.) and extinction measurement (Ext.) wavelengths. (**b**) Hysteresis-like behavior of the measured extinction at 693 nm (left axis) and of the resonant wavelength of the dipolar resonance (right axis), extracted from (**a**) as a function of temperature. (**c**) Extinction of the VO_2_ nanodisk array measured at 693 nm upon continuous illumination by a continuous wave laser (633 nm) with gradually increasing power. Adapted with permission from [121]. Copyright 2016 American Chemical Society. Lower panel (**d**–**f**): Tunable GST-plasmonic nanohole array metasurface. The MWIR optical input is imaged through GST-PNA filer in its initial, amorphous state (**d**), with an initial center wavelength, λ_1_. Through a laser pulse (532 nm) incident on the GST-PNA active area, the GST crystallinity is modified (phase change) and the resultant transmission response is spectrally shifted (**e**). (**f**) Pump energy controls the GST state, which in turn changes its refractive index, thus spectrally shifting the center wavelength from the resonant PNA. A “reset pulse” returns the GST to its initial state, thus resetting the device to initial transmission center wavelength. Adapted from [124] under the terms of Creative Commons CC BY licence.

Laser-induced optical tuning of a metasurface consisting of VO_2_ nanodisks (270 nm in diameter and 200 nm in height) on a fused silica substrate was characterized by Kepič and colleagues [121]. The demonstration of light-triggered phase transition tuning of the VO_2_ nanodisk arrays was performed using a 633 nm continuous wave laser, which drives the VO_2_ phase transition with the consequent extinction modulation of the visible Mie resonances with a modulation of 1.5 dB in the visible spectral range between 400 and 900 nm (see Figure 8a, Upper Panel).

Optical stimuli can also be used to tune the chiro-optical response that derives from the interactions of circularly polarized light with the metasurface.

Zheng and colleagues proposed an all-silicon chiral metasurface based on “Z-shaped” micropillars to produce highly efficient circular polarization differential transmittance (CPDT) in the Terahertz (in the range of 0.8–2 THz) [122]. The static CPDT and the dynamic chiral behaviors of the proposed metasurface can be modulated by optical pumping at 1064 nm. The selective transmission characteristics of left circularly polarized (LCP) and right circularly polarized (RCP) are realized by adjusting the silicon conductivity, where higher conductivity values lead to lower transmittance of the THz radiation. This kind of optical modulation could find many potential applications in photon-spin selective devices, such as circularly polarized light detectors and chiral sensors.

Hu et al. experimentally demonstrated an anisotropic plasmon-induced transparency (PIT) metasurface performing efficient photoswitching of THz radiation and polarization multiplexed temporal dynamics [123]. The metasurface is composed of a fourfold symmetric coupled resonator between metallic split rings (SRRs) and closed-ring resonators (CRRs), and it is designed to generate an isotropic Fano-type resonance. Then, two semiconductors, i.e., crystalline Si and amorphous Ge, are embedded into the gaps of SRRs aligned along two orthogonal directions. The temporal modulation of THz radiation polarization is obtained through optical tuning by femtosecond pump pulse at 800 nm. The optical pump modulation generates photocarriers with a relaxation time difference of three orders of magnitude between layer I (crystalline silicon) and layer II (amorphous germanium). The optically induced switching dynamic can be alternated between a quasi-steady state with a recovery time larger than 2 ns for the x-polarized incidence and an ultrafast transient state with a recovery time <25 ps in the orthogonal polarization channels. This kind of metasurface could find application in the field of switchable metamaterial devices.

Moreover, in the MWIR, optically tunable metasurfaces were proposed by Julian and coworkers, who presented a phase change tunable metasurface acting as a spectral filter based on GST-embedded plasmonic nanohole arrays (PNAs); see Figure 8d,e. This metasurface showed high transmission efficiency, narrowband performance, continuous tuning range, and fully reversible operation [124]. Reversible optical tunability is achieved through a single nanosecond laser pulse (532 nm), which induces a phase change in GST crystallinity toward its amorphous phase, changing its refractive index, thus spectrally shifting the center wavelength. Through this mechanism, this metasurface can act as an optical tunable spectral filter with high transmittance (∼70%) in the 3–5 µm range and as a near perfect reflector off-resonance. A reset laser pulse guides GST to its initial state, thus resetting the device to its initial transmission center wavelength; see Figure 8f.

### 2.6. Multiple Stimuli Tuning

In recent years, metasurfaces that can be modulated by multiple external stimuli at the same time have been proposed. This new frontier has allowed the simulation and design of demonstrators with new functionalities or with extreme fine-tuning capabilities. Here, we propose three very interesting works that are opening this frontier.

**Figure 9:**
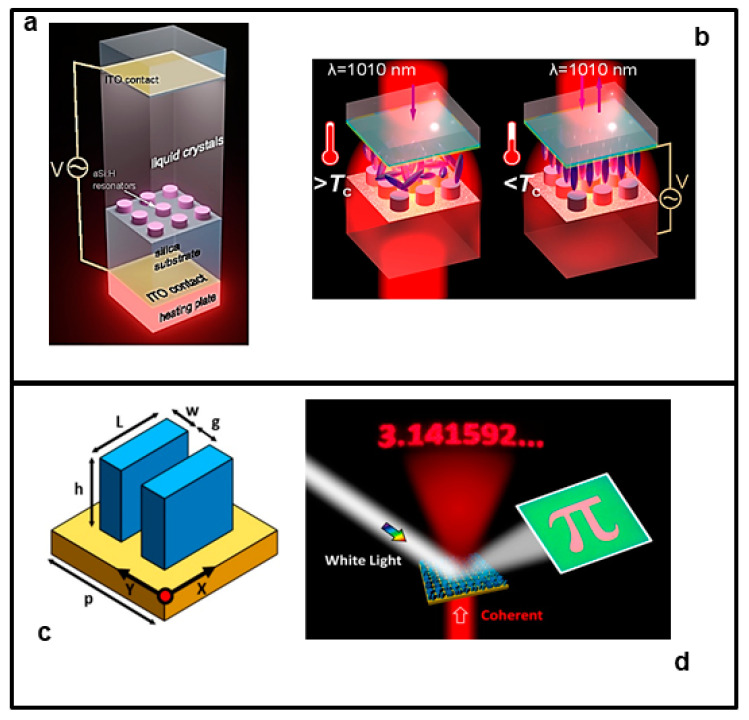
Example of two multi-stimuli tunable metasurfaces. Upper panel (**a**,**b**): (**a**) Schematic illustration of the metasurface cell. (**b**) System configurations for polarization-independent tuning for a LC metasurface with (left) LCs in its isotropic phase and with (right) an applied voltage. Figure 9a,b adapted with permission from [125]. Copyright 2016 American Chemical Society. Lower panel (**c**,**d**): (**c**) Illustration of the unit cell, which has two identical nanoantennas: length L = 300 nm, width W = 100 nm or 50 nm, and gap g = 100 nm. (**d**) Operation schematic of the crypto-display: the transmission mode uses single-wavelength coherent light to produce the holographic image of “3.141592...” in the image plane, whereas the reflection mode uses white light to represent a reflected coloured image of “π”. Figure 9c,d adapted with permission from [127]. Copyright 2018 American Chemical Society.

Chengjun Zou et al. [125] experimentally studied a dual-stimuli tuning LC-integrated dielectric metasurface that combines both electrical and thermal tunability in the spectral window of 600–750 nm. The mestasurface is composed of an ITO-coated glass decorated with 1 μm high Mie-resonant silicon cylinders integrated into a liquid crystal cell. They observed large resonance shifts in transmittance by simultaneous application of voltage and temperature stimuli, allowing to enter tuning regimes inaccessible by application of a single stimulus only. In particular, they demonstrated that the simultaneous double stimulus allows fine tuning of modulation depths, nearly-polarization-independent tuning, and gated response, all of which cannot be achieved with just a single stimulus. The structure and functioning of this metasurface is shown in Figure 9a,b.

Chengjun Zou et al. [126] demonstrated multi-modular metasurfaces made by combining highly resonant, asymmetrical metasurfaces with light- and temperature-sensitive polymeric materials at near-infrared wavelengths. They have shown that the tunability of the metasurface response is reversible with respect to light alone, to temperature alone, and to the application of both stimuli. The system is composed of a unit cell repeated in the x- and y-direction with a period of 770 nm. Several quasi-bound states in the continuum modes are generated through symmetry breaks in the unit cell. The nanostructures consist of silicon nanobars with slightly different lengths (L_1_ = 380 nm and L_2_ = 350 nm) on an SiO_2_ substrate. The nanobars have a center-to-center distance of 360 nm, which is smaller than a half period (385 nm). The nanobar height is 280 nm and its width is 200 nm. Finally, we have a 500 nm P(TEGA-co-AZO) polymer coating layer. Thanks to this multitunable approach, maximum spectral resonance shifts of almost double the full width at half maximum and accompanied by over 60% absolute modulation in transmittance were obtained. The metasurface transmittance spectra were calculated and measured in the range of 1100–1500 nm. In general, this article introduces the realization of multifunctional metadevices able to adapt to a complex environment, showing that multiresponsive hybrid systems could be promising candidates for multidimensional sensing applications.

Yoon et al. [127] experimentally demonstrated a crypto-display composed of two kinds of silicon nanoantennas with two operating modes that do not influence each other. This device works in the visible spectral range. The response of their system can be modulated differently if the system is subjected to white light or coherent light. In fact, when illuminated with white light, the metasurface behaves like a normal reflective display; instead, when illuminated with single wavelength coherent light, the metasurface generates a hologram that reveals the encrypted phase information (see Figure 9c,d). This study paves the way for developments of new safety devices.

## 3. Remarks and Future Perspectives

The meta-optics research field has experienced an exponential growth of theoretical and experimental research papers over the past few years. Metasurfaces present several benefits for modern optical technologies. In particular, they allow the miniaturization of optical components, reducing the size, weight, and power (SWaP) requirements for optical systems. Furthermore, they enable smart functions that are not readily achievable with conventional optics and offer the possibility of real-time optical re-configurability thanks to their nanoscale dimensionality. This technological landscape has attracted industry interest for numerous applications such as machine autonomy, human vision through augmented reality technologies, smart vision, new machine–human interfaces, and compact instrumentation for space applications.

The development of tunable metasurfaces whose optical properties can be tailored using an external stimulus will further boost the range of possible applications. Indeed, in conventional optical systems, the adjustment of optical parameters (such as focal length, steering angle, and transmission) is achieved by mechanical means, by physically changing the position or the orientation of one or more components in the optical system. This requires not only the realization of a system with moving parts, but also the implementation of mechanical actuators (e.g., electric motors or piezoelectric elements).

The use of tunable metalenses can change this approach, allowing not only to obtain an adjustment of the optical properties without movable parts, but also to obtain adjustment times orders of magnitude faster than those achievable with macroscopic mechanical actuators, and with a fraction of the required power.

To meet the above-mentioned challenges, research frontiers have focused on several fundamental and applied directions that simplify metasurfaces’ design for specific applications. These include the implementation of deep learning and inverse design approaches, as discussed in the Introduction. Another challenge is the possibility of achieving metasurface tunability on a pixel level; in this regard, phase change materials represent a promising approach. A further exciting research frontier is the application of optical metasurfaces for quantum light generation, detection, and classification.

From the analysis of the literature presented in this review, it appears that most of the current research focuses on electrical tuning (see Section 2.1) and mechanical tuning (Section 2.5) of metasurfaces. This is understandable because electrical tuning would allow an easy integration of the metasurface with mass production methods used for microelectronics, such as CMOS technology. Moreover, electrical tuning can allow high modulation speed/frequency, at least for some classes of materials described here, i.e., semiconductors and materials with an electro-optical response.

The interest in mechanical tuning approaches is motivated in particular by the availability of technical solutions derived from the technology of micro-mechanical actuators (e.g., MEMS), which provide a convenient platform in view of the miniaturization and integration of devices. On the other hand, the scalability of these devices can present issues.

Moreover, optoelectronics can take the advantages of meta-optics for the precision control of excitonic excitations and for the complete spatio-temporal wavefront control in general.

The development of novel meta-optical components is particularly important for detecting infrared light, especially in the medium wavelength and long wavelength IR, where the current detectors are still not very efficient. Metasurface-enhanced infrared detectors can indeed find important applications in night vision and the spectral identification of chemical compounds.

Most of the approaches treated in this review deal with globally tunable metasurfaces, i.e., metasurfaces whose optical properties are changed across the whole extension of the component.

On the other hand, very interesting developments would be allowed by the achievement of metasurface tunability at the level of the individual meta-atom. This would in principle allow complete control of a metasurface response, enabling the full modulation of the wavefront (in terms of amplitude, polarization, and propagation direction) at the level of the single cell. Prerequisites for such an achievement are not only the development of materials with widely tunable optical properties, but also the development of convenient methods to achieve random access actuation of the individual meta-atoms.

In this sense, PCMs such as GST are very promising, as these materials feature fast switching time, broad modulation of the optical properties, and response to different kinds of stimuli (which eventually results in a temperature-driven phase change). For instance, the fact that PCMs can respond to optical stimuli (via local heating) already provides a way to obtain spatially modulated tuning of the meta-atoms’ properties across the metasurface extension, by projecting on it a suitable light intensity distribution. These approaches can pave the way toward the development of “universal” metasurfaces (that is, metasurfaces that can accomplish several different functions by addressing the response of the individual meta-atoms); for instance, highly efficient, ultracompact active optical elements such as tunable lenses, spatial light modulators, and addressable polarizing optics.

Undoubtedly, the development of metasurfaces reconfigurable at the level of individual meta-atoms is the next milestone in the research on tunable meta-optics.

Another interesting field that is currently expanding is that of non-linear tunable metasurfaces. Non-linear optical effects, such as frequency conversion, all-optical modulation, generation of non-classical light, cascaded harmonic generation, asymmetric frequency conversion, and the nonlinear chiral response, are very desirable for many applications in optics.

In this frame, advances in tunable resonant nonlinear metasurfaces will also allow to achieve full all-optical control over the transient behavior of nonlinearities at femtosecond switching speeds.

In particular, time-modulated metasurfaces can be employed for dynamic wavefront engineering and space–time photonics, enabling next-generation photonic technologies for applications such as nanoscale pulse shapers, optical switches, and so on.

A further growing field is that of tunable metasurfaces for chemical and biological sensing. In particular, a topic of great interest is that of metasurfaces functionalized with receptors for specific molecular targets. Indeed, sensors based on functionalized metasurfaces can pave the way towards emerging applications such as portable medical diagnostics, efficient detection of single molecules, or environmental monitoring. The tuning of the functionalized metasurface response will improve the resolution between possible interfering species, enhancing sensors’ selectivity and sensitivity. Recently, the potential use of tunable metasurfaces for the integration of bioelectronics with tissues, as well as the design of devices for wireless health monitoring and therapy, have also been proposed. Obviously, further accurate testing will be required for medical use.

## Data Availability

No new data were created or analyzed in this study. Data sharing is not applicable to this article.
